# High-dose eicosapentaenoic acid (EPA) improves attention and vigilance in children and adolescents with attention deficit hyperactivity disorder (ADHD) and low endogenous EPA levels

**DOI:** 10.1038/s41398-019-0633-0

**Published:** 2019-11-20

**Authors:** Jane Pei-Chen Chang, Kuan-Pin Su, Valeria Mondelli, Senthil Kumaran Satyanarayanan, Hui-Ting Yang, Yi-Ju Chiang, Hui-Ting Chen, Carmine M. Pariante

**Affiliations:** 1 Department of Psychological Medicine, Institute of Psychiatry, Psychology and Neuroscience, King’s College London, London, WC2R 2LS UK; 2 Department of Psychiatry, China Medical University Hospital, Taichung, Taiwan; 3 College of Medicine, China Medical University, Taichung, Taiwan; 4 College of Nutrition, Taipei Medical University, Taipei, Taiwan

**Keywords:** Pathogenesis, Learning and memory

## Abstract

No studies have examined the relationship between endogenous polyunsaturated fatty acids (PUFAs) levels and treatment response to PUFAs. We conducted a 12-week, double-blind, placebo-controlled trial comparing the effects of high-dose eicosapentaenoic acid (EPA, 1.2 g) and placebo on cognitive function (continuous performance test) in *n* *=* 92 youth (age 6–18-years-old) with Attention Deficit Hyperactivity Disorder (ADHD). Blood erythrocytes PUFAs were measured before and after treatment, to examine the effects of baseline endogenous EPA levels on treatment response and the effects of EPA treatment on PUFAs levels. Secondary measures included other ADHD symptoms, emotional symptoms, and levels of plasma high-sensitivity c-reactive protein (hs-CRP) and brain-derived neurotrophic factor (BDNF). Overall, EPA group improved more than placebo group on focused attention (variability, Effect size (ES) = 0.38, *p* = 0.041); moreover, within youth with the lowest baseline endogenous EPA levels, EPA group improved more than placebo group in another measure of focused attention (hit reaction time, HRT, ES = 0.89, *p* = 0.015) and in vigilance (HRT interstimulus interval changes, HRTISIC, ES = 0.83, *p* = 0.036). Interestingly, EPA group improved *less* than placebo group in impulsivity (commission errors), both overall and in youth with the *highest* baseline EPA levels, who also showed less improvement in other ADHD and emotional symptoms. EPA increased blood erythrocytes EPA by 1.6-fold but not DHA levels, and did not affect hs-CRP and BDNF plasma levels. In conclusion, EPA treatment improves cognitive symptoms in ADHD youth, especially if they have a low baseline endogenous EPA level, while youth with high EPA levels may be negatively affected by this treatment.

## Introduction

A deficiency of omega-3 polyunsaturated fatty acids (n-3 PUFAs) may play a role in the pathogenesis of attention deficit hyperactivity disorder (ADHD)^1^. N-3 PUFAs, including eicosapentaenoic acid (EPA) and docosahexaenoic acid (DHA), are essential fatty acids (EFA) for our brain and body^2^, and have been closely associated with cognitive function^3^ and academic performance^4^. Our recent meta-analysis has shown that youth with ADHD have lower blood levels of DHA, EPA and total n-3 PUFAs, when compared with typically developing youth^1^. Moreover, EFA deficiency (a measure of symptoms such as excessive thirst, dry skin, brittle nails and small skin bumps) has been described in children with ADHD^5^, and the severity of EFA deficiency has been associated with ADHD symptom severity^6^.

Although meta-analysis report that stimulants are tolerated and effective treatments for youth with ADHD, these drugs have side effects that need monitoring, such as insomnia, risk of abuse, and changes in blood pressure, heart rate and body weight^7–10^. N-3 PUFAs have a very good tolerability and safety profile, and thus may be a preferable treatment option for youth with ADHD. Indeed, our recent meta-analysis has shown that n-3 PUFAs treatment improves clinical symptoms in youth with ADHD^1^, although individual studies have conflicting findings, possibly because of different dosages of EPA and DHA used across studies. Of note, a previous meta-analysis examining clinical trials in children with ADHD showed that a high dose of omega-3 (1–2 g) supplementation was required to show significant improvement in clinical symptoms^11^. Moreover, the therapeutic effects of n-3 PUFAs in ADHD seems to be particularly evident when measuring cognitive function (for example, attention and impulsivity) rather than generic ADHD symptoms^1^. Interestingly, most of the n-3 PUFAs studies in ADHD used either a high-dose DHA^12^, or a relative low-doses of DHA and EPA combination treatment^13^, despite the evidence that EPA is the more effective therapeutic component, at least in depression^14–16^. In fact, our meta-analysis^1^ shows that only studies with EPA supplementation greater or equal to 500 mg per day find an improvement in the clinical hyperactivity-impulsivity symptoms.

Of note, there is some evidence that psychiatric treatments may be more effective when subjects are stratified based on biomarkers that are mechanistically relevant to the intervention. For example, two studies have shown that depressed patients with higher inflammation, as measured by C-reactive protein (CRP) or high interleukin (IL)-6, have a better response to the anti-inflammatory, infliximab, or to n-3 PUFAs supplementation, respectively^17,18^. Although there have been no clinical trials using PUFAs’ endogenous levels to stratify patients in PUFAs clinical trials, we have previously shown a direct link between lower endogenous PUFAs levels and increased risk of developing interferon-alpha (IFN-α)-induced depression, suggesting that endogenous PUFAs levels can offer clinically-relevant information on course and outcome ^19^.

Finally, in term of the possible mechanism underpinning the therapeutic effects of PUFAs in ADHD, both inflammation and neuroplasticity have been proposed as relevant. There is some evidence suggesting that ADHD is associated with increased inflammation: for example, children with ADHD are more likely to suffer from asthma and atopic dermatitis^20,21^, and two studies have found increased interleukin IL-6 in children with ADHD^22,23^. In addition, neurotrophins, such as brain-derived neurotrophic factor (BDNF), may also play a role in ADHD, although the results have been inconsistent^24^. PUFAs are generally considered to modulate neuroplasticity^25^ and to be anti-inflammatory^26^; in fact we have previously shown that pre-treatment with EPA reduces the onset of depression induced by the pro-inflammatory cytokine, IFN-α^16^. Thus, both these biological systems might be relevant to the mechanisms of action of PUFAs in ADHD.

Based on the gaps in knowledge described above, we have conducted a 12-week double-blind, randomised, placebo-controlled trial (RCT) in youth (6–18 years) with ADHD, which: (1) compares a high dose of EPA (1.2 g/day) with placebo; (2) measures cognitive function as the primary outcome; (3) examines endogenous PUFAs levels to stratify patients and to examine the effects of treatments on PUFAs levels; and (4) investigate the inflammatory biomarker, high-sensitivity CRP (hs-CRP), and the neurotrophin, BDNF, as potential mechanisms.

## Patients and methods

### Participants

The Institutional Review Board of China Medical University Hospital (CMUH, Taichung, Taiwan) approved this study (CMUH 104-REC-058) and written informed consent was obtained from the participants and their parents. We recruited youth aged 6–18 years, with the Diagnostic and Statistical Manual of Mental Disorders, Fifth Edition (DSM-5) diagnosis of ADHD with either inattention (attention deficit disorder, ADD), hyperactivity, or combined presentation; we also assessed the presence of oppositional defiant disorder (ODD), characterised by symptoms such as often losing temper, arguing with adults, defying rules and blaming others. All diagnoses were confirmed by a child and adolescent psychiatrist, and all participants were referred to the Department of Psychiatry, CMUH, from July 2016 to December 2017. The participants were either drug naïve or had no medication for the past 6 months. The ClinicalTrials.gov identifier for this study is: NCT03542643.

The exclusion criteria were: (1) Intelligence quotient <70, based on a documented history of mental retardation; (2) for those ages 6–12 years old, a Peabody Picture Vocabulary Test-Revised (PPVT-R) percentile scores less than 5% (indicating speech delay or intellectual disability); (3) other comorbid psychiatric disorders, such as autism spectrum disorder, anxiety disorder, conduct disorder, and other major psychiatric disorders; (4) comorbid physical disorders, such as thyroid dysfunction and cerebral palsy; (5) currently using n-3 PUFAs supplements; and (6) allergy to n-3 PUFAs.

**Fig. 1:**
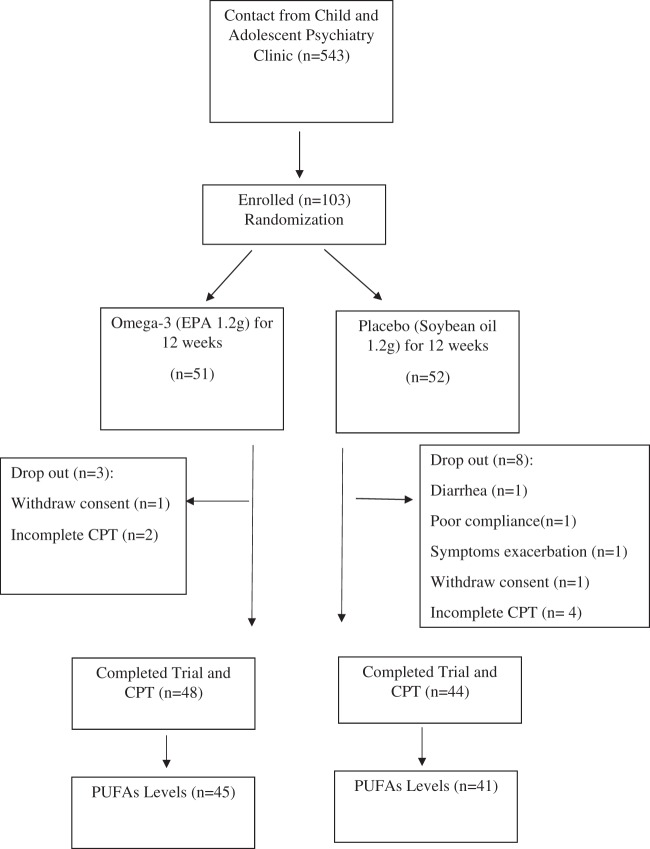
Flow chart of study recruitment. CPT continuous performance test, EPA eicosapentaenoic acid, n number, PUFAs polyunsaturated fatty acids

We performed power calculations for the sample size of the RCT. The ES for n-3 PUFAs in ADHD is around 0.2–0.3, but when we excluded the studies with less strictly diagnosis and those with low concentrations of EPA (less than 500 mg).We were to find the effect size of high dose EPA treatment is around 0.38 to 0.81^1^. The online sample size calculator is used to calculate the sample size, https://www.ai-therapy.com/psychology-statistics/sample-size-calculator. Significance level: 0.05, power: 0.8, ES: 0.38 to 0.81, and the estimated sample sizes are between *n* = 246 to *n* = 50. One hundred and three youth were recruited and randomised to n-3 PUFAs (1.2 g/day EPA) or placebo (1.2 g/day soybean oil) for 12 weeks, and 92 subjects (mean age 9.49 + 3.05 years, 85.9% male) completed the 12-week trial (see Fig. 1). The randomisation numbers were generated from the computer. The investigators were blinded to both the group allocation during the study and when assessing the outcome measurements.

Our main outcome measures were the four items of the Continuous Performance Test (CPT), measuring focused attention, impulsivity, sustained attention and vigilance, assessed at baseline and week 12 (end of the trial). Additional measures assessed at baseline and week 12 were the Strength and Difficulties Questionnaire (SDQ), for the emotional problems of the child, and the Digit Span Subset of Wechsler Intelligence Scale for Children-Fourth Edition (WISC-IV) for working memory and short-term memory; moreover, we used the Swanson, Nolan, and Pelham IV (SNAP-IV) to measure ADHD clinical symptoms rated by parents, teachers and youth (for those ages 12 years or older), at baseline and week 1, 2, 4, 8, and 12. Blood samples were collected at baseline and week 12 (end of the trial) for evaluation of erythrocytes levels of PUFAs (measured using gas chromatography, samples available in *n* = 86) and levels of plasma hs-CRP (*n* = 80) and BDNF (*n* = 84), using enzyme-linked immunosorbent assay (ELISA). Additional details of the clinical, cognitive and laboratory measurements are provided in the Supplementary File.

All the main analyses were conducted on the 92 subjects who completed the trial, as the primary outcome measure (the CPT) was only collected at baseline and at the end of the trial. Reassuringly, the 11 children who dropped out from the trial were not different in ADHD severity from the 92 who completed, although they tended to be younger (see Supplementary Table S1). Youth who had both CPT and baseline endogenous EPA measurements were further stratified in groups based on tertiles of EPA levels (EPA ≤ 0.91%, *n* = 29; >0.91 to 1.08%, *n* = 30; and EPA > 1.08%, *n* = 27).

### Statistical analyses

All statistical analysis was carried out with Statistical Package for the Social Science (SPSS), version 25.0 for Windows^27^. For all analyses, we compared youth on placebo with youth on EPA for changes in scores (cognitive and symptoms) and levels of biomarkers, between baseline and 12-weeks; that is, we compared the deltas, using t-test for deltas that were normally distributed (most of the variables) and Mann–Whitney test for the deltas that were not normally distributed (that is, variability, HRT block changes (HRTBC), SDQ emotion problems (SDQE), SDQ conduct problems (SDQC), SDQ peer problems (SDQP), WISC-IV digit span forward (WISCDSF) and longest digit span forward (WISCLDSF)). This same approach was used after stratification of subjects based on endogenous EPA levels. In addition, effect size (ES) and 95% confidence interval (CI)s were calculated, when appropriate. A *p*-value of <0.05 indicates statistical significance.

## Results

### Compared with the placebo group, the EPA group improves more in focused attention but less in impulsivity

Overall (*n* = 92), we found that youth on EPA improved more than youth on placebo in focused attention (CPT variability, ES = 0.38, CI = −0.05 to 0.80, *p* = 0.041) but less than those on placebo in impulsivity (commission errors, EF = −0.43, CIs = −0.84 to −0.01, *p* = 0.025). There were no other differences between the groups in other CPT measures of focused attention (omission errors, *p* = 0.615; HRT standard deviation (HRTSD), *p* = 0.573; detection *p* = 0.094), impulsivity (HRT, *p* = 0.059; perseveration, *p* = 0.604), sustained attention (HRTBC, *p* = 0.650) and vigilance (HRT interstimulus interval changes (HRTISIC), *p* = 0.492) (see Table 1).

**Table 1:** The CPT scores at week 12 from baseline between the EPA and placebo groups

Mean (SD)	EPA (*n* = 48)			Placebo (*n* = 44)			*P*
	Baseline	Wk12	Changes	Baseline	Wk12	Changes	
d’	−1.27 (0.92)	−1.41 (1.15)	−0.14 (0.63)	−1.23 (0.85)	−1.60 (0.98)	−0.36 (0.63)	0.094
OM	0.09 (0.08)	0.08 (0.10)	−0.01 (0.09)	0.07 (0.07)	0.07 (0.07)	0.00 (0.06)	0.615
COM	0.53 (0.16)	0.49 (0.20)	−0.04 (0.15)	0.58 (0.16)	0.48 (0.18)	−0.10 (0.13)	0.025*
PER	0.04 (0.05)	0.04 (0.03)	−0.01 (0.03)	0.04 (0.05)	0.03 (0.03)	−0.01 (0.04)	0.604
HRT	509.40 (117.91)	210.74 (117.99)	1.34 (61.87)	481.72 (107.89)	508.06 (119.67)	26.34 (63.44)	0.059
HRTSD	299.20 (166.61)	293.10 (164.84)	−6.10 (108.48)	266.26 (156.23)	274.11 (148.29)	7.85 (128.01)	0.573
VAR	139.21 (98.08)	133.81 (88.80)	−15.44 (83.50)	117.36 (81.98)	132.91 (86.45)	14.15 (72.19)	**0.041**^#^*
HRTBC	11.08 (25.58)	15.57 (29.62)	4.49 (45.50)	12.01 (17.98)	14.21 (17.95)	2.21 (22.22)	0.650^#^
HRTISIC	73.93 (45.66)	73.32 (44.38)	−0.61 (44.05)	63.61 (51.51)	69.05 (51.47)	5.44 (39.84)	0.492

*Changes* indicates the changes in scores from baseline at week 12, *COM* commission error, *CPT* continuous performance test, *d’* detection, *EPA* eicosapentaenoic acids, *HRT* hit reaction time, *HRTSD* HRT standard deviation, *VAR* variability, *HRTBC* HRT block change, *HRTISIC* HRT interstimulus interval change, *n* number; *PER* perseveration, *SD* standard deviation, *wk* week. The *p* values are from Independent-sample *t*-test, unless Mann–Whitney test result

^#^Mann–Whitney test results

Asterisk (*) indicates a statistical significance of *p* < 0.05

There were no significant differences in the overall samples between EPA and placebo groups for changes in memory (see Supplementary Table S2) and in clinical (Supplementary Table S3) and emotional (Supplementary Table S4) symptoms.

As 51 out of 92 participants had comorbid ODD, and children with ADHD and ODD tend to be generally less responsive to pharmacological treatment^28^, we tested whether this improvement in attention was present in a subgroup analysis of these 51 participants; and indeed, we found that, as in the whole group, youth on EPA improved more than youth on placebo in focused attention (*ES* = 0.66, *CI* = 0.07 to 1.23, *p* = 0.025).

### EPA-induced changes are influenced by stratification on baseline endogenous EPA levels

**Fig. 2:**
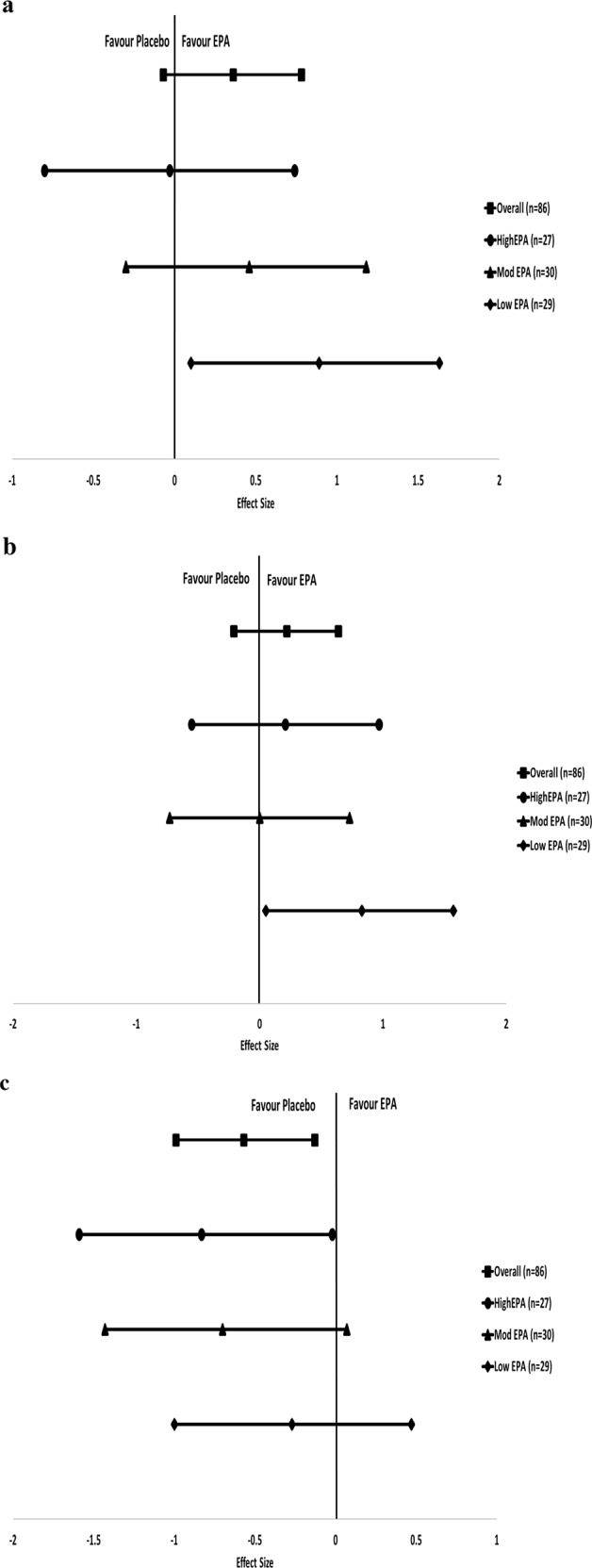
The effect size of EPA on cognitive function, (a) HRT, (b) HRTISIC and (c) COM, with stratification of baseline EPA levels. **a** EPA have a greater effect on HRT than placebo in the Low EPA group, with an effect size of 0.89, the confidence intervals of 0.10 to 1.63, *p* = 0.015. There were no differences between the n-3 PUFAs group and placebo group on HRT of CPT in the overall, High EPA and Mod EPA group. **b** EPA have a greater effect on HRTISIC than placebo in the Low EPA group, with an effect size of 0.83, the confidence intervals of 0.05 to 1.57, *p* = 0.036. There were no differences between the n-3 PUFAs group and placebo group on HRT of CPT in the overall, High EPA and Mod EPA group. **c** The placebo group improved more on the commission errors than the placebo group in the overall, with an effect size of −0.43, the confidence intervals of −0.84 to −0.01, *p* = 0.025, and high EPA group, with an effect size of −0.83, the confidence intervals of −1.59 to −0.02, *p* = 0.022. There were no differences between the EPA group and placebo group on COM of CPT in the Mod EPA and Low EPA group. The *x*-axis is the effect size. Note, COM, commission errors; EPA, eicosapentaenoic acid; High EPA, EPA > 1.08%; HRT, hit reaction time; HRTISIC, HRT interstimulus interval change; Mod EPA, 0.91% < EPA < 1.08%; Low EPA, EPA < 0.91%; *n* number

Baseline EPA levels are presented in Table 2. After stratification based on tertiles of baseline EPA (*n* = 86), we found that, in the *lowest* EPA group, youth on EPA *improved* more than youth on placebo in another measure of attention (HRT, ES = 0.89, CIs = 0.10 to 1.63, *p* = 0.015; see Fig. 2a) and in vigilance (HRTISIC, ES = 0.83, Cis = 0.05 to 1.57, *p* = 0.036, see Fig. 2b). Interestingly, we also found that, in the *highest* endogenous EPA group, youth on EPA improved *less* than those on placebo in impulsivity (commission error; ES = −0.83, CIs = −1.59 to −0.02, *p* = 0.022, Fig. 2c). There were no other differences between placebo and EPA groups on other CPT measures (see Supplementary Tables S5–S7).

**Table 2:** The levels of PUFAs at week 12 from baseline between the EPA and placebo groups

Mean (SD)	EPA (*n* = 45)			Placebo (*n* = 41)			*P*
	Baseline	Wk12	Changes	Baseline	Wk12	Changes	
AA (%)	9.91 (1.95)	9.23 (2.54)	−0.68 (3.02)	9.30 (2.93)	8.50 (3.47)	−0.79 (4.44)	0.887
DHA (%)	3.68 (0.98)	3.59 (1.27)	−0.10 (1.49)	3.49 (1.27)	3.10 (1.42)	−0.45 (1.97)	0.342
EPA (%)	1.18 (0.63)	1.95 (1.12)	0.77 (1.12)	1.06 (0.37)	0.91 (0.40)	−0.15 (0.62)	<0.0001****
Total n-3 (%)	5.56 (1.25)	5.98 (2.10)	0.48 (2.30)	5.29 (1.55)	4.49 (1.79)	−0.87 (2.58)	0.012*
Total n-6 (%)	27.58 (3.57)	25.80 (4.50)	−1.78 (5.80)	26.56 (5.69)	24.78 (6.43)	−1.78 (8.40)	0.999
N-6/n-3 ratio	5.17 (1.07)	4.76 (1.58)	−0.43 (1.83)	5.24 (0.97)	6.23 (2.32)	0.99 (2.73)	0.008**^#^
hs-CRP (*n* = 43vs37)	2.00 (0.90)	1.98 (0.81)	−0.04 (0.38)	2.27 (1.39)	2.10 (1.15)	−0.10 (0.87)	0.747
BDNF (*n* = 44vs40)	756.07 (394.99)	74.43 (330.54)	−681.64 (344.42)	771.00 (371.30)	−59.33 (289.54)	−824.80 (370.54)	0.070

*AA* arachidonic acid, *BDNF* brain-derived neurotrophic factor, *Changes* indicates the changes of levels from baseline at week 12, *DHA* docosahexaenoic acid, *EPA* eicosapentaenoic acid, *hs-CRP* high-sensitivity c-reactive protein, *n* number, *n-3* omega-3 polyunsaturated fatty acids, *n-6* omega-6 polyunsaturated fatty acids, *SD* standard deviation, *wk* week

Asterisk (*) indicates a statistical significance of *p* < 0.05

Asterisks (**) indicates a statistical significance of *p* < 0.01

Asterisks (****) indicates a statistical significance of *p* < 0.0001. The *p* values are from Independent-sample *t*-test, unless *X*^2^ test or Mann–Whitney test result

^#^Mann–Whitney test results

^a^*X*^2^ test results

In terms of secondary analyses, we also found that, in the *highest* EPA group, youth on EPA improved *less* than those on placebo in parental reports of inattention (SNpA), oppositional symptoms (SNpO), total ADHD symptoms (SNpH) and internalising problems (SDQIn), as well as in teachers’ report of inattention (SNtaA) (see Supplementary Tables S8–S10).

### EPA treatment increases EPA, but not DHA levels, and does not affect hs-CRP and BDNF

As expected, EPA group showed a greater increase in EPA (*p* < 0.0001) and total n-3 PUFAs levels (*p* *=* 0.012), and a greater decrease of n-6/n-3 ratio (*p* = 0.008), compared with placebo (see Table 2). However, surprisingly, there was no difference in changes in DHA levels between EPA and placebo group (*p* = 0.342). Changes in hs-CRP and BNDF values are also reported in Table 2; there were no differences between EPA and the placebo (*p* = 0.747 and *p* = 0.070, respectively).

Changes in these biological measures after stratification based on tertiles are presented in Supplementary Tables S11–S13. Across the three subgroups, EPA levels always increase in the EPA group. Again, across the three groups, there were no differences between EPA and placebo groups for changes in hs-CRP and BDNF.

## Discussion

We have compared the effects of EPA and placebo on cognitive and clinical symptoms of ADHD and on mechanistically-related biomarkers. Overall, EPA improves (more than placebo) focused attention at the CPT test, one of our main outcome measures. Moreover, youth with low endogenous EPA levels show even stronger effects of EPA in a further measure of attention (HRT) and in a measure of vigilance (HRTISIC), both improving more than in the placebo group. To our knowledge, this is the first study to report the effects of EPA monotherapy in ADHD, and the first study ever to use endogenous baseline PUFAs levels to stratify subjects in a PUFAs clinical trial. Further analyses indicate that EPA treatment in those with *high* endogenous EPA levels may actually have detrimental effects.

Our main findings are consistent with (at least some) previous studies investigating n-3 PUFAs (usually, combinations of EPA and DHA) in ADHD. Our recently-published meta-analysis^1^ has shown that PUFAs improves attention and impulsivity in children with ADHD, but the results of the individual studies have been inconsistent. For example, Sinn et al.^29^ found an improvement in attention, but Voigt et al.^30^ did not; similarly, Vaisman et al.^31^ found an improvement in impulsivity, but Voigt et al.^30^ found that, much like this present study, placebo did better than PUFAs for impulsivity. While it is difficult to draw firm conclusions on the reasons behind these discrepant findings, it is worth mentioning that both our study and Voigt et al.^30^ have around half of the sample with comorbid ODD. Interestingly, subgroup analyses in our data show that the improvement in attention does occur in the children with comorbid ODD, thus suggesting that specific classes of symptoms (impulsivity vs. attention, in this case) may respond differently to EPA also based on the presence of comorbid psychiatric conditions.

To test whether low endogenous PUFAs levels predict response to PUFAs, we use a similar approach to that used by two previous ‘personalised psychiatry’ studies, i.e., Raison et al.^17^ and Rapaport et al.^18^ : they both found antidepressant effects by an anti-TNF-alpha medication and by n-3 PUFAs, but only in patients with high baseline inflammation, i.e., those with biological evidence of abnormalities in the mechanism targeted by the interventions (in these two studies, inflammation). Consistent with this framework, we find that youth with the lowest levels of baseline endogenous EPA show the largest improvement in cognitive function following EPA. Although there are no other similar studies, previous research, in healthy children, as well as children and adults with ADHD and other neurodevelopmental disorders, has shown that PUFAs improve cognitive function *more* in the presence of low “n-3 PUFAs status”, i.e., in those with evidence of low dietary intake of fish, or if they present symptoms of the aforementioned EFA deficiency^32^.

Interestingly, and much like our study, Raison et al.^17^ and Rapaport et al.^18^ find not only that high inflammation predicts a better response to the anti-inflammatory intervention, but also that low inflammation predicts a better response to placebo, i.e., lowering inflammation in people who already have low inflammation actually has adverse effects. We also find that children with the *highest* baseline EPA levels perform *better* on placebo than on EPA, in impulsivity and in parental and teachers’ reports of ADHD and emotional symptoms. A previous meta-analysis in depression has also shown a “J-shaped curve” in the protective effects of PUFAs, increasing for doses of up to 1.8 g/day of n-3 PUFAs (or 0.6 g/day of EPA + DHA intake), and then decreasing for higher doses^33^; this suggests that very high doses of PUFAs (or, by extensions, normal doses in people with high endogenous PUFAs levels), may have adverse effects. However, it is important to emphasise that this J-shaped curve has been described for the intake of PUFAs from the diet, rather than supplementation^33^, and hence some potential mechanisms to explain these negative effects (high dietary intake of omega-6, or environmental contaminants like mercury from fish) are not present in our study. Reassuringly, most studies of children with ADHD (conducted largely in Western countries) have shown average endogenous EPA levels that are lower than those in our study^1^, probably because of the low-fish and high-omega-6 dietary intake in non-Asian countries^34^; thus, it is unlikely that non-Asian children would normally reach endogenous PUFAs levels that are similar to those in the highest tertile of our sample. Nevertheless, with fish intake and other natural sources of omega-3 being constantly advocated as part of a healthy diet, it is important to be aware that supplementing those who already have high levels of EPA may be detrimental.

The youth receiving EPA in our study show an increase in blood erythrocytes EPA and total n-3 PUFAs levels, and a decrease in the n-6/n-3 ratio, at the end of the 12 weeks. This is an expected finding, and it confirms the compliance of the youth with treatment. However, we also found that DHA levels do not increase in youth who received EPA, which is surprising since EPA is physiologically converted into DHA. However, this finding is consistent with a previous study showing that 1 g/day EPA in patients with depression only increases plasma EPA levels but not DHA levels^35^. Similarly, studies in patients with dyslipidemia and healthy subjects found that 3-4 g/day EPA increases plasma EPA but not DHA levels^36,37^. In humans, there is a poor enzymatic conversion of EPA to DHA^37^, and genetic variants in fatty acid desaturase 2 gene (*FADS2*), the key enzyme responsible for the conversion from EPA to DHA, further reduce this conversion^38^. Interestingly, and of specific interest for our study, a single nucleotide polymorphism (SNP) in *FADS2* has been significantly associated with ADHD^39^, suggesting that the genetic profile of ADHD may have further effects on PUFAs metabolism. While we wanted specifically to test whether EPA alone was enough (following from the evidence in depression^16^), most of the other studies included in our previous meta-analysis^1^ have combined EPA + DHA interventions, and, indeed, in a previous cross-over clinical trial, an increase in erythrocyte EPA and DHA after a combined EPA + DHA intervention was associated with improved attention and behavioural symptoms in children with ADHD^40^. Thus, taken together, our data do seem to suggest that, for ADHD clinical symptoms, combined EPA and DHA may have a broader impact than EPA alone on clinical symptoms, possibly because affecting both EPA and DHA levels.

We investigated inflammation as one mechanism that might explain the actions of EPA on cognitive function in ADHD. EPA has been shown to have anti-inflammatory action via antagonising membrane arachidonic acid (AA) formation and inhibition of the synthesis of pro-inflammatory mediators^41^. However, EPA did not affect hs-CRP levels in our study. Of course, the average hs-CRP levels of youth with ADHD in our study (just above 2 mg/L) were below the threshold for even low-grade inflammation (3 mg/L); thus, the presence of only very mild levels of inflammation might have made impossible to detect an anti-inflammatory action of EPA. Moreover, it is also important to highlight that a previous study found a significant decrease in plasma CRP levels in children with ADHD treated with *combined* 900 mg PUFAs per day (DHA 165 + EPA 635 mg)^42^; thus, it is also possible that EPA monotherapy is ineffective to induce an anti-inflammatory action. In a previous study we found that EPA pre-treatment is able to prevent inflammation-induced depression following IFN-alpha treatment^16^; however, we did not measure CRP or indeed other immune biomarkers in that study, and thus we cannot exclude that the beneficial effects of EPA were due to changes in other mechanisms. Thus, the present study does support the possibility that both EPA and DHA are needed to exert a clear anti-inflammatory action.

The levels of neurotrophins, BDNF, were also not affected by EPA in this study. Previous studies have demonstrated that treatments for ADHD, such as methylphenidate^43^ and atomoxetine^44^, modulate levels of BDNF, although BDNF levels were increased after 6 weeks treatment of methylphenidate^43^ and decreased after a 3-month treatment of atomoxetine^44^. Studies of the associations between n-3 PUFAs intake and BDNF levels have also been inconsistent: for example, a positive association has been reported between n-3 PUFAs consumption and serum BDNF levels in adolescents^45^, but clinical trials do not find an effect of n-3 PUFAs on BDNF levels in adults with distress following trauma^46^ or in adults with diabetes mellitus and depression^47^. Again, preclinical evidence indicates that neuroplasticity effects of PUFAs may require both EPA and DHA^48^, suggesting an explanation for our negative findings.

Our study, although with several strengths, is not without possible limitations. First the ADHD population is heterogeneous, with age ranging 6–18 years and comorbidity with ODD; however, these characteristics make our sample more similar to the ADHD population in clinical settings, which reinforces the generalisability of our study. A second limitation is the duration of our study, 12 weeks, while most of the studies included in our previous meta-analysis^1^ have longer durations (mean of 15.6 weeks). Perhaps a longer duration of the trial would have been more successful in eliciting effects of EPA on clinical symptoms in ADHD. However, longer trials risk high drop-out rates, and we wanted to test an intervention that was easily deliverable in non-specialist settings; our drop-out rate of around 10% testifies to the success of this approach. It is also interesting to note that those who discontinued the study had a younger age than those who completed the study; this may be due to the fact that parents of young children with ADHD might feel less distressed by the symptoms and thus less keen in pursuing treatment, as suggested by previous studies^49^. Finally, we did not perform multiple comparison corrections in our analysis; we felt that this would have been too stringent, and might have generated false-negative findings. Instead, we have relied on the presentation of effect size differences throughout the papers, so that clinical significance, rather than statistical, is used to convey the strengths of the results; improvement in the overall group was at least moderate in its effect size (.38), and the improvements in the youth with the lowest EPA levels were big in their effect sizes (>0.8), thus we trust that these are true positive findings.

In conclusion, our study shows some benefits of EPA monotherapy on cognitive symptoms of ADHD. As amply discussed, it is possible that a combined EPA + DHA strategy might have been more beneficial, and as such we support the recent recommendation by a panel of ADHD experts that patients who prefer omega-3 supplements over stimulants should take a combination of DHA and EPA at doses ≥750 mg per day for at least 12 weeks^50^. However, we additionally recommend that this strategy should be even more strongly advocated for children with evidence of low endogenous PUFAs levels, as indicated by direct measurement, dietary habits or symptoms of EFA deficiency. Conversely, in the cases where high endogenous levels of PUFAs might already be present because of a dedicated diet or previous supplements, PUFAs levels should be investigated before trialling this strategy, to limit any potential negative effects. In this way, we can start bringing the benefits of ‘personalised treatment’ to children with ADHD.

## Supplementary information

Supplementary Material
