# Supplementary material for: High-dose eicosapentaenoic acid (EPA) improves attention and vigilance in children and adolescents with attention deficit hyperactivity disorder (ADHD) and low endogenous EPA levels
Source: Transl Psychiatry. 2019 Nov 20;9:303. doi: 10.1038/s41398-019-0633-0 (PMC6864068; doi:10.1038/s41398-019-0633-0)
Supplement: Supplementary file 1 — Supplementary Material [file 41398_2019_633_MOESM1_ESM.docx]

**Supplementary Information**

**High-Dose Eicosapentaenoic Acid (EPA) Improve Attention and Vigilance**

**in Children and Adolescents with Attention Deficit Hyperactivity Disorder (ADHD)**

**and Low EPA Levels**

**Running title**: EPA Improve Attention in ADHD

Jane Pei-Chen Chang^1,2,3^, Kuan-Pin Su^1,2,3*^, Valeria Mondelli^1^, Senthil Kumaran Satyanarayanan^2,3^, Hui-Ting Yang^4^, Yi-Ju Chiang^2,3^, Hui-Ting Chen^2,3^,

Carmine M. Pariante^1^

^1^Department of Psychological Medicine, Institute of Psychiatry, Psychology and Neuroscience, King’s College London, London, UK

^2^Department of Psychiatry, China Medical University Hospital, Taichung, Taiwan

^3^College of Medicine, China Medical University, Taichung, Taiwan

^4^College of Nutrition, Taipei Medical University, Taipei, Taiwan

***Correspondence:**

Kuan-Pin Su, Department of Psychiatry & Mind-Body Interface Laboratory (MBI-Lab),

China Medical University Hospital & College of Medicine, China Medical University, No. 2 Yu-Der Rd, North District, Taichung 404, Taiwan, cobolsu@gmail.com

**SUPPLEMENTARY METHODS**

**Measures**

***Chinese version of the Swanson, Nolan, and Pelham, Version IV Scale (SNAP-IV) Questionnaires***

Parents, teachers and children older than or equal to 12 years old were asked to report attention deficit hyperactivity disorder (ADHD) severity of the child with the SNAP-IV.^1-4^ The parent, teacher and youth versions of SNAP-IV have been validated elsewhere.^1-3^ SNAP-IV consists of 18 Diagnostic and Statistical Manual of Mental Disorders (DSM)-5 symptoms of ADHD, including inattention (items 1–9), hyperactivity (items 10–15), impulsivity (items 16–18), and symptoms of the criteria for the oppositional defiant disorder (ODD) (items 19–26). The sum of scores of items 1-18 indicates the total ADHD score. The symptom severity of each symptom item was rated on a 4-point rating scale (0 = not at all, 1 = just a little, 2 = quite a bit, and 3 = very much). A higher total score on items 1–18 indicated a higher severity of ADHD symptoms. The ADHD group is further stratified by subtypes: inattention subtype (IA) is defined by SNAP-IV inattention subscale (IAs) > 12, the hyperactivity-impulsivity subtype (HI) is defined by SNAP-IV hyperactivity-impulsivity subscale (HIs) > 12, the combined subtype is defined by both SNPAP-IV IAs and HIs > 12, and subthreshold ADHD is defined by neither SNAP-IV IAs nor HIs score >12. ODD is defined with an SNPA-IV ODD score > 13.

**Strength and Difficulty Questionnaire (SDQ)**

The parents reported on emotional problems of the child with the SDQ. The SDQ is a behavioural screening questionnaire for children and adolescents age 3-16 years old, and consists of 5 domains: emotional problems, conduct problems, hyperactivity, peer problems and prosocial behavior.^5^ Each domain is composed of 5 items and each item is scored on a 3-point rating scale (0 = not true, 1 = somewhat true, 2 = certainly true). A higher score for emotional symptoms, conduct problems, hyperactivity, peer relationship problems, and a total of the four domains, represent total difficulties. On the other hand, a lower score for prosocial behavior subscale indicates a problem. The internalizing problems score is comprised of the scores of emotional and peer problems domains. The externalizing problems score is comprised of the scores of the conduct problems and hyperactivity domains. The reliability and validity of the Chinese version of the SDQ have been reported elsewhere.^6^

**Cognitive Assessment**

***Peabody Picture Vocabulary Test-Revised (PPVT-R)***

Receptive vocabulary knowledge was measured with the PPVT-R^7,8^ to screen for intellectual disability in children age 6 to 12 years of age at baseline (scores less than 5% may indicate speech delay or intellectual disability). For this task, children were shown sets of four pictures and asked to point to the picture representing the word spoken by the examiner. The PPVT-R manual provides normative data that were collected from a sample of 886 typically developing (TD) children with normal hearing from 3 to 12 years of age. The standard scores for this test were derived from the raw scores using a scale provided by the test developers. The standard scores were based on a mean of 100 and a standard deviation (SD) of 15.

***Conners’ Continuous Performance Test (CPT )***

The CPT is a 14-minute computerized task for children aged 8 years and older.^9^ It requires the individual to tap on the spacebar when any character except “X” is shown on the screen. There are 6 blocks in the CPT, including 3 sub-blocks that each contain 20 letter presentations, for a total of 360 trials. The sub-blocks differ in interstimulus intervals (ISI) of 1, 2 and 4 s, and the sequence of ISI conditions is presented randomly. The domains of CPT performances include omission errors (the number of times not responding to a target), commission errors (the number of times responding to a nontarget), hit reaction time (HRT, the period of time between the presentation of the stimulus and the response), variability (intraindividual variability in HRT), perseveration (HRT<100 milliseconds (ms)), detection (d’, the ability to discriminate between targets and nontargets), HRT standard deviation (HRTSD, consistency of RT), HRT changed by blocks (HRTBC, the slope of changes in RT over 6 blocks as the test progresses), HRT changed by ISIs (HRTISIC, whether performance decreases with longer ISIs). These domains can be grouped into 4 dimensions: focused attention, impulsivity, sustained attention, and vigilance.^10^ Focused attention includes omission errors, HRTSD, variability, and detection; impulsivity includes commission errors, HRT and perseveration; sustained attention includes HRTBC; and vigilance includes HRTISIC.

***Wechsler Intelligence Scale-Fourth Edition (WISC-IV) Digit Span Subtest***

The Digit Span assessment is a measure of short-term memory for children aged 7 and over.^11^ There two parts to the Memory for Digit Span assessment: Digits Forward and Digits Backward. Each tap distinct but interdependent cognitive functions. Digit Forward primarily taps short-term memory, while Digits Span Backward measures the ability of the child to manipulate verbal information while in temporary storage. The higher the points indicate a better memory function of the child.

**Laboratory Assessments**

***Blood Inflammatory and neurotrophins biomarker levels***

Blood samples were obtained in the morning (0800h to1000h) after 12-hour fasting. Venous blood were extracted into 5mL K2 ethylenediaminetetraacetic acid tubes (BD, Franklin Lakes, NJ, USA) and were centrifuged at 1200 x g for 10 min (25°C) and the plasma were stored at -80°C until further analysis. Patients enrolled in this study were assessed for inflammatory markers including concentrations of high-sensitivity c-reactive protein (hs-CRP) (DuoSet ELISA Development, R&D Systems, Inc., USA), and neurotrophins including brain-derived neurotrophic factor (BDNF) (DuoSet ELISA Development, R&D Systems, Inc., USA) by enzyme-linked immunosorbent assay (ELISA) method. The samples were analysed in duplicate according to the manufacturer's instructions. The neurotrophin (BDNF) levels were expressed in picogram per millilitre (pg/mL) and the plasma hs-CRP levels were expressed in milligram per litre (mg/L).

***Blood polyunsaturated fatty acids (PUFAs) levels***

The fatty acid composition of erythrocyte membranes was analyzed by thin-layer chromatography and the level of individual fatty acid was measured with gas chromatography of methyl esters (Lipid Standards, FAME, Sigma Co., St. Louis, MO, USA). Fatty acid profiles were identified by comparing the retention times with those of appropriate standard fatty acid methyl esters. The detailed step-by-step procedures have been published and described elsewhere.^12^ The levels of each fatty acid were expressed as a percentage of total fatty acids. Laboratory measures were conducted on coded samples by workers who were blind to the information about the subjects.

SUPPLEMENTARY RESULTS

**Memory Function**

There was no difference in the changes in the forward memory (WISC longest digit span forward, WISCLDSF, *p* = .943; WISC digit span forward, WISCDSF, *p* =; .867), backward memory (WISC longest digit span backward, WISCLDSB, *p* = .252; WISC digit span backward, WISCDSB, *p* = .073) and total memory scores (WISCDS Total, *p* = .125) at 12 weeks from baseline between n-3 PUFAs and placebo group (Supplementary Table 2).

**Clinical Symptoms**

There was no difference in the changes of parent-, teacher- and self-reported inattention (*p* = .072, .066, .628), hyperactivity (*p* = .075, .766, .628) or ODD (*p* = .207, .759, .418) subscale scores and total ADHD scores (teacher- and self-reported only; *p* = .170, .923) of SNAP-IV at 12 weeks from baseline between n-3 PUFAs and placebo group (Supplementary Table 3); the placebo group had a reduction in the parent-reported total ADHD scores at week 12 (*p* = .042).

**Emotional symptoms**

There were no differences in the changes of the SDQ Emotion (*p* = .476), Conduct (*p* = .885), Hyperactivity (*p* = .471), Peer Problems (*p* = .487), Prosocial Behaviour (*p* = .386), Externalizing Problems (*p* = .676) and Internalizing Problems (*p* = .238) subscale scores between the n-3 PUFAs and placebo group (Supplementary Table 4).

SUPPLEMENTARY REFERENCES

1. Gau, S.S. Psychometric properties of the Chinese version of the Swanson, Nolan, and Pelham, Version IV Scale-Teacher Form. *J Pediatr Psychol* 2009; 34(8): 850-861.
2. Gau, S.S. Psychometric properties of the Chinese version of the Swanson, Nolan, and Pelham, version IV Scale-Parentform.*Int J Methods Psychiatr Res* 2008; 18(1): 35-44.
3. Nichols, J.Q. et al. Exploration of the factor structure of ADHD in adolescence through self, parent, and teacher reports of symptomatology. *J Abnorm Child Psycho*l 2017; 45(1): 35-44.
4. Swanson, J., Posner, M., Fusella, J., Wasdell, M., Sommer, T., Fan, J. Genes and attention deficit hyperactivity disorder. *Curr Psychiatry Rep* 2001; 3(2): 92-100.
5. Goodman R. The Strengths and Difficulties Questionnaire: a research note. *J Child Psychol Psychiatry* 1997; 38(5): 581-586.
6. Liu, S.K. et al. Psychometric properties of the Chinese version of Strength and Difficulties Questionnaire. *Compr Psychiatry* 2013; 54(6): 720-730.
7. Wu, C.M. et al. Long-term language levels and reading skills in mandarin-speaking prelingually deaf children with cochlear implants. *Audiol Neurootol* 2011; 16(6): 359-380.
8. Dunn, L.M., Hottel, J.V. Peabody Picture Vocabulary Test performance of trainable mentally retarded children. A*m J Ment Deficit* 1961; 65: 448-452.
9. Conners, C.K. *Conners’ Continuous Performance Test III: Computer program for Windows technical guide and software manual*. Multi-Health Systems: North Tonwanda, NY, 2014.
10. Egeland, J., Kovalik-Gran, I. Validity of the factor structure of Conners’ CPT. *J Atten Disord* 2010; 13(4): 347-57.
11. Wechsler, D. *The Wechsler Intelligence Scale for Children-Fourth Edition*. Pearson: London, 2003.
12. Chiu,C.C. et al. Polyunsaturated fatty acid deficit in patients with bipolar mania. *Eur Neuropsychopharmacol* 2003; 13(2): 99-103.

**SUPPLEMENTARY TABLES**

Supplementary Table S1: The Demographic and clinical characteristics of those who completed the study and of those who dropped out

| Mean(SD) | Included  (n=92) | Dropped out  (n=11) | *P* |
| --- | --- | --- | --- |
| Age (yrs) | 9.49(3.05) | 7.64(2.06) | .053 |
| Gender (male, %) | 79(85.9) | 10(90.9) | 1.000^a^ |
| SNpA | 17.39(5.50) | 17(5.81) | .825 |
| SNpH | 14.41(6.45) | 15.73(5.10) | .516 |
| SNpO | 12.46(5.47) | 13.64(6.49) | .509 |
| SNpAH | 31.80(10.06) | 32.73(9.45) | .773 |

Note: n, number; SD, standard deviation; SNAP-IV, Swanson, Nolan and Pelham (SNAP) Questionnaire-Fourth Edition; SNpA, inattentive subscale score of parent version of SNAP-IV at baseline; SNpAH, total ADHD score of parent version of SNAP-IV at baseline; SNpH, hyperactivity-impulsivity subscale score of parent version of SNAP-IV at baseline; SNpO, oppositional defiant disorder subscale score of parent version of SNAP-IV at baseline; yrs, years. The p values are from Independent-sample t-test, unless X^2^ test result. ^a^X^2^ test results.

Supplementary Table S2: The changes in the WISCDS scores at week 12 from baseline between the EPA and placebo groups

| **Mean (SD)** | **EPA (n=48)** | | | **Placebo (n=44)** | | | ***P*** |
| --- | --- | --- | --- | --- | --- | --- | --- |
|  | **Baseline** | **Wk12** | **Changes** | **Baseline** | **Wk12** | **Changes** |  |
| WISCLDSF | 8.04(1.32) | 8.56(.71) | .52(1.18) | 8.14(1.07) | 8.55(.79) | .41(.87) | .943^#^ |
| WISCDSF | 13.15(2.52) | 14.31(1.56) | 1.17(2.24) | 13.61(2.22) | 14.57(1.87) | .95(1.48) | .867^#^ |
| WISCLDSB | 3.94(1.36) | 4.54(1.58) | .60(1.07) | 4.20(1.65) | 4.52(1.72) | .32(1.31) | .252 |
| WISCDSB | 6.92(2.45) | 7.94(2.66) | 1.02(1.44) | 7.23(3.06) | 7.59(2.82) | .36(2.01) | .073 |
| WISCDS Total | 20.6(3.83) | 22.25(3.52) | 2.19(2.67) | 20.84(4.60) | 22.16(4.11) | 1.32(2.71) | .125 |

Note: EPA, eicosapentaenoic acids; n, number; SD, standard deviation; WISC, Weschler Intelligence Scale for Children-Fourth Edition; WISCDS, WISC Digit Span; WISCDSB, WISC Digit Span Backward; WISCDSF, WISC Digit Span Forward scores; WISCDS Total, total score of WISC Digit Span subtest; WISCLDSB, WISC Longest Digit Span Backward scores; WISCLDSF WISC Longest Digit Span Forward scores. The *p* values are from Independent-sample t-test, unless [Mann-Whitney](https://blog.minitab.com/blog/adventures-in-statistics/best-way-to-analyze-likert-item-data%3A-two-sample-t-test-versus-mann-whitney) test result. ^#^ [Mann-Whitney](https://blog.minitab.com/blog/adventures-in-statistics/best-way-to-analyze-likert-item-data%3A-two-sample-t-test-versus-mann-whitney) test results.

Supplementary Table S3: The changes of clinical ADHD symptoms at week 12 from baseline between the EPA and placebo groups

| **Mean(SD)** | **EPA** | | | **Placebo** | | |  |
| --- | --- | --- | --- | --- | --- | --- | --- |
| **Parent Reports** | **(n=48)** | | | **(n=44)** | | | ***P*** |
|  | **Baseline** | **Wk12** | **Changes** | **Baseline** | **Wk12** | **Changes** |  |
| SNpA | 16.96(5.58) | 14.75(5.34) | -2.21 (6.08) | 17.86(5.43) | 13.50(5.63) | -4.36 (5.19) | .072 |
| SNpH | 14.29(6.77) | 12.13(5.86) | -2.17 (4.84) | 14.55(6.16) | 10.50(5.50) | -4.04 (5.15) | .075 |
| SNpO | 12.21(6.03) | 10.44(6.03) | -1.77 (5.04) | 12.73(4.85) | 9.55(5.44) | -3.18 (5.62) | .207 |
| SNpAH | 31.25(10.22) | 26.88(10.02) | -4.27 (9.51) | 32.41(9.97) | 24.00(10.17) | -8.41 (9.19) | .042* |
| **Teacher Reports** | **(n=44)** | | | **(n=39)** | | | ***P*** |
| SNtaA | 16.02(7.32) | 16.09(7.06) | .07(4.44) | 15.31(4.51) | 13.7(4.86) | -1.46 (2.69) | .066 |
| SNtaH | 14.18(8.06) | 12.86(7.55) | -1.32 (3.74) | 11.51(6.89) | 9.95(6.20) | 1.54(2.85) | .766 |
| SNtaO | 9.86(7.74) | 9.07(6.82) | -.80 (4.30) | 9.46(6.67) | 8.93(6.18) | -.54(3.13) | .759 |
| SNtaAH | 30.20(13.26) | 28.95(13.32) | -1.25 (6.76) | 26.82(9.82) | 23.65(9.50) | -3.00 (4.34) | .170 |
| **Youth Self-Reports** | **(n=12)** | | | **(n=10)** | | | ***P*** |
| SNsA | 15.23(4.49) | 11.17(6.51) | -4.17 (5.32) | 13.90(6.14) | 10.09(3.99) | -2.90 (5.38) | .628^#^ |
| SNsH | 9.46(6.36) | 6.33(5.10) | -3.92 (5.62) | 11.70(7.87) | 6.55(5.59) | -4.60 (7.09) | .628^#^ |
| SNsO | 10.54(5.61) | 7.58(6.17) | -3.58 (3.65) | 11.70(6.58) | 6.73(5.76) | -4.30 (6.07) | .418^#^ |
| SNsAH | 24.69(9.08) | 18.33(11.75) | -7.25  (10.04) | 25.60(13.39) | 16.64(8.48) | -7.50  (11.74) | .923^#^ |

Note: : EPA, eicosapentaenoic acids; n, number; SD, standard deviation; SNAP-IV, Swanson, Nolan and Pelham (SNAP) Questionnaire-Fourth Edition; SNpA, inattentive subscale score of parent version of SNAP-IV; SNpAH, total ADHD score of parent version of SNAP-IV; SNpH, hyperactivity-impulsivity subscale score of parent version of SNAP-IV; SNpO, oppositional defiant disorder subscale score of parent version of SNAP-IV; SNsA, inattentive subscale score of youth version of SNAP-IV; SNsAH, total ADHD score of youth version of SNAP-IV; SNsH, hyperactivity-impulsivity subscale score of youth version of SNAP-IV; SNsO, oppositional defiant disorder subscale score of youth version of SNAP-IV; SNtaA, inattentive subscale score of teacher version of SNAP-IV; SNtaAH, total ADHD score of teacher version of SNAP-IV; SNtaH, hyperactivity-impulsivity subscale score of teacher version of SNAP-IV; SNtaO, oppositional defiant disorder subscale score of teacher version of SNAP-IV. The *p* values are from Independent-sample t-test, unless [Mann-Whitney](https://blog.minitab.com/blog/adventures-in-statistics/best-way-to-analyze-likert-item-data%3A-two-sample-t-test-versus-mann-whitney) test result. ^#^ [Mann-Whitney](https://blog.minitab.com/blog/adventures-in-statistics/best-way-to-analyze-likert-item-data%3A-two-sample-t-test-versus-mann-whitney) test results. * indicates a statistical significance of *p <* .05.

Supplementary Table S4: The changes in the SDQ scores at week 12 from baseline between the EPA and placebo groups

| **Mean(SD)** | **EPA (n=48)** | | | **Placebo (n=44)** | | | ***P*** |
| --- | --- | --- | --- | --- | --- | --- | --- |
|  | **Baseline** | **Wk12** | **Changes** | **Baseline** | **Wk12** | **Changes** |  |
| SDQE | 3.48(2.01) | 2.94(2.21) | -.54(1.64) | 3.50(1.90) | 2.70(1.79) | -.79(1.79) | .476^#^ |
| SDQC | 4.35(2.27) | 3.63(2.04) | -.73(1.43) | 4.02(2.12) | 3.34(1.92) | -.68(1.58) | .885^#^ |
| SDQH | 8.04(1.77) | 7.13(2.25) | -.92(2.17) | 7.95(1.64) | 6.75(1.71) | -1.20(1.56) | .471 |
| SDQP | 4.00(2.15) | 3.92(2.04) | -.08(1.71) | 4.18(2.14) | 3.75(2.20) | -.43(1.40) | .487^#^ |
| SDQPB | 6.15(2.47) | 6.58(2.20) | .44(1.72) | 6.34(2.00) | 6.48(2.01) | .14(1.58) | .386 |
| SDQEx | 12.40(3.51) | 10.75(3.44) | -1.65(2.90) | 11.98(2.92 | 10.09(2.99) | -1.89(2.57) | .676 |
| SDQIn | 7.48(3.13) | 6.85(3.30) | -.63(2.59) | 7.68(3.16) | 6.45(3.32) | -1.23(2.24) | .238 |

Note: EPA, eicosapentaenoic acids; n, number; SD, standard deviation; SDQ, Strength and Difficulty Questionnaire; SDQE, SDQ Emotion; SDQC, SDQ Conduct; SDQH, SDQ Hyperactivity; SDQP, SDQ Peer Problems; SDQPB, SDQ Prosocial Behaviour; SDQEx, SDQ Externalizing Problems; SDQIn, SDQ Internalizing Problems; yrs, years. The *p* values are from Independent-sample t-test, unless [Mann-Whitney](https://blog.minitab.com/blog/adventures-in-statistics/best-way-to-analyze-likert-item-data%3A-two-sample-t-test-versus-mann-whitney) test result. ^#^ [Mann-Whitney](https://blog.minitab.com/blog/adventures-in-statistics/best-way-to-analyze-likert-item-data%3A-two-sample-t-test-versus-mann-whitney) test results.

Supplementary Table S5: The changes in cognitive measures of the low EPA tertile group after 12 weeks

| Mean (SD) | EPA (n=13) | Placebo (n=16) | *P* |
| --- | --- | --- | --- |
| d’ | -.11(.75) | -.13(.57) | .948 |
| OM | -.03(.04) | -.01(.04) | .068 |
| COM | -.02(.19) | -.06(.10) | .846 |
| PER | -.00(.02) | .01(.03) | .449 |
| HRT | -28.89(43.26) | 14.06(46.17) | .015* |
| HRTSD | -23.13(8.55) | 28.83(94.24) | .056 |
| VAR | -22.40(105.65) | 38.71(53.30) | .065 |
| HRTBC | -9.07(22.74) | 2.58(21.21) | .249 |
| HRTISIC | -13.07(25.32) | 7.69(24.57) | .036* |
| WISC-IV | (n=13) | (n=16) | *P* |
| WISCLDSF | .62(1.26) | .25(.58) | .475 |
| WISCDSF | 1.23(2.05) | .50(1.15) | .423 |
| WISCLDSB | .46(1.13) | .63(1.15) | .812 |
| WISCDSB | .69(1.11) | .94(1.57) | 1.000 |
| WISCDS Total | 1.92(2.78) | 1.44(2.48) | .812 |

Note: COM, commission error; CPT, continuous performance test; d’, detection; EPA, eicosapentaenoic acids; HRT, hit reaction time; HRTSD, HRT standard deviation; HRTBC, HRT block change; HRTISIC, HRT interstimulus interval change; low EPA tertile, EPA < .91%; n, number; PER, perseveration; SD, standard deviation; VAR, variability; WISC, Weschler Intelligence Scale for Children-Fourth Edition; WISCDS, WISC Digit Span; WISCDSB, WISC Digit Span Backward; WISCDSF, WISC Digit Span Forward scores; WISCDS Total, total score of WISC Digit Span subtest; WISCLDSB, WISC Longest Digit Span Backward scores; WISCLDSF WISC Longest Digit Span Forward scores. The *p* values are from [Mann-Whitney](https://blog.minitab.com/blog/adventures-in-statistics/best-way-to-analyze-likert-item-data%3A-two-sample-t-test-versus-mann-whitney) test result. * indicates a statistical significance of *p* < .05

**Supplementary Table S6: The changes in cognitive measures of the intermediate EPA tertile group after 12 weeks**

| Mean (SD) | EPA (n=18) | Placebo (n=12) | *P* |
| --- | --- | --- | --- |
| d’ | -.24(.64) | -.48(.57) | .200 |
| OM | -.01(.08) | .02(.05) | .095 |
| COM | -.03(.14) | -.14(.18) | .087 |
| PER | -.02(.03) | -.03(.14) | .249 |
| HRT | 9.64(82.00) | 45.97(75.84) | .215 |
| HRTSD | -8.59(120.04) | -8.68(107.59) | .692 |
| VAR | -16.29(73.10) | -14.70(70.60) | .444 |
| HRTBC | -2.45(17.78) | 6.46(19.56) | .215 |
| HRTISIC | 8.68(52.75) | 9.21(46.30) | .950 |
| WISC-IV | (n=18) | (n=12) | *P* |
| WISCLDSF | .28(.57) | .67(.98) | .325 |
| WISCDSF | .56(1.42) | 1.67(1.78) | .087 |
| WISCLDSB | .89(1.18) | .08(1.56) | .079 |
| WISCDSB | 1.39(1.75) | 1.67(2.59) | .095 |
| WISCDS Total | 1.94(2.36) | 1.83(3.33) | .662 |

Note: COM, commission error; CPT, continuous performance test; d’, detection; EPA, eicosapentaenoic acids; HRT, hit reaction time; HRTSD, HRT standard deviation; HRTBC, HRT block change; HRTISIC, HRT interstimulus interval change; intermediate EPA tertile, .91% < EPA < 1.08%; N, number; PER, perseveration; SD, standard deviation; VAR, variability; WISC, Weschler Intelligence Scale for Children-Fourth Edition; WISCDS, WISC Digit Span; WISCDSB, WISC Digit Span Backward; WISCDSF, WISC Digit Span Forward scores; WISCDS Total, total score of WISC Digit Span subtest; WISCLDSB, WISC Longest Digit Span Backward scores; WISCLDSF WISC Longest Digit Span Forward scores. The *p* values are from [Mann-Whitney](https://blog.minitab.com/blog/adventures-in-statistics/best-way-to-analyze-likert-item-data%3A-two-sample-t-test-versus-mann-whitney) test result.

**Supplementary Table S7: The changes in cognitive measures of the high EPA tertile group after 12 weeks**

| Mean (SD) |  |  |  |
| --- | --- | --- | --- |
| CPT | EPA (n=14) | Placebo (n=13) | *P* |
| d’ | -.13(.54) | -.57(.73) | .085 |
| OM | -.02(.07) | -.02(.08) | .943 |
| COM | -.03(.14) | -.14(.11) | .022* |
| PER | -.01(.03) | -.01(.04) | .943 |
| HRT | 14.48(45.03) | 12.57(68.70) | .616 |
| HRTSD | -15.65(93.76) | -5.58(188.76) | .720 |
| VAR | -16.19(84.73) | 13.31(93.85) | .713 |
| HRTBC | 4.26(20.63) | -1.12(25.47) | .583 |
| HRTISIC | -11.19(40.95) | -1.24(51.58) | .202 |
| WISC-IV | (n=14) | (n=13) | *P* |
| WISCLDSF | .79(1.72) | .38(1.04) | .756 |
| WISCDSF | 1.93(3.22) | .77(1.30) | .550 |
| WISCLDSB | .57(.85) | .15(1.14) | .220 |
| WISCDSB | 1.00(1.30) | -.23(2.01) | .054 |
| WISCDS Total | 2.93(3.17) | .54(2.50) | .061 |

Note: COM, commission error; CPT, continuous performance test; d’, detection; EPA, eicosapentaenoic acids; HRT, hit reaction time; HRTSD, HRT standard deviation; HRTBC, HRT block change; HRTISIC, HRT interstimulus interval change; high EPA tertile, EPA > 1.08%; n, number; PER, perseveration; SD, standard deviation; VAR, variability; WISC, Weschler Intelligence Scale for Children-Fourth Edition; WISCDS, WISC Digit Span; WISCDSB, WISC Digit Span Backward; WISCDSF, WISC Digit Span Forward scores; WISCDS Total, total score of WISC Digit Span subtest; WISCLDSB, WISC Longest Digit Span Backward scores; WISCLDSF WISC Longest Digit Span Forward scores. The *p* values are from [Mann-Whitney](https://blog.minitab.com/blog/adventures-in-statistics/best-way-to-analyze-likert-item-data%3A-two-sample-t-test-versus-mann-whitney) test result. * indicates a statistical significance of *p* < .05.

**Supplementary Table S8: The changes in clinical measures of the low EPA tertile group after 12 weeks**

| Mean (SD) | EPA | Placebo |  |
| --- | --- | --- | --- |
| Parent Reports | (n=13) | (n=16) | *P* |
| SNpA | -1.92(7.03) | -3.31(3.57) | .650 |
| SNpH | -2.54(5.64) | -2.44(4.70) | .812 |
| SNpO | -1.77(6.23) | -1.81(5.66) | .682 |
| SNpAH | -4.46(11.79) | -5.75(7.02) | .779 |
| Teacher Reports | (n=11) | (n=14) | *P* |
| SNtaA | -.36(6.93) | -.79(2.01) | .936 |
| SNtaH | -2.55(4.91) | -1.29(2.16) | .936 |
| SNtaO | -1.18(6.26) | -.43(2.14) | .809 |
| SNtaAH | -2.91(10.21) | -2.07(1.98) | .767 |
| Youth Self-Reports | (n=4) | (n=4) | *P* |
| SNsA | -4.25(7.93) | -4.75(6.40) | 1.000 |
| SNsH | 1.50(2.08) | -4.50(11.12) | .343 |
| SNsO | -4.5(3.70) | -6.00(5.94) | .486 |
| SNsAH | -.25(13.67) | -9.25(17.45) | .486 |
| SDQ | (n=13) | (n=16) | *P* |
| SDQE | -.62(1.33) | -.44(1.46) | .779 |
| SDQC | -.31(1.18) | -.13(2.06) | .914 |
| SDQH | -1.23(2.49) | -.94(1.34) | 1.000 |
| SDQP | -.23(1.24) | .12(1.31) | .475 |
| SDQPB | .62(1.85) | .13(1.78) | .650 |
| SDQEx | -1.54(3.50) | -1.06(2.69) | .948 |
| SDQIn | -.85(2.19) | -.31(2.02) | .682 |

Note: EPA, eicosapentaenoic acids; low EPA tertile, EPA < .91%; n, number; SD, standard deviation; SDQ, Strength and Difficulty Questionnaire; SDQE, SDQ Emotion; SDQC, SDQ Conduct; SDQH, SDQ Hyperactivity; SDQP, SDQ Peer Problems; SDQPB, SDQ Prosocial Behaviour; SDQEx, SDQ Externalizing Problems; SDQIn, SDQ Internalizing Problems; SNAP-IV, Swanson, Nolan and Pelham (SNAP) Questionnaire-Fourth Edition; SNpA, inattentive subscale score of parent version of SNAP-IV; SNpAH, total ADHD score of parent version of SNAP-IV; SNpH, hyperactivity-impulsivity subscale score of parent version of SNAP-IV; SNpO, oppositional defiant disorder subscale score of parent version of SNAP-IV; SNsA, inattentive subscale score of youth version of SNAP-IV; SNsAH, total ADHD score of youth version of SNAP-IV; SNsH, hyperactivity-impulsivity subscale score of youth version of SNAP-IV; SNsO, oppositional defiant disorder subscale score of youth version of SNAP-IV; SNtaA, inattentive subscale score of teacher version of SNAP-IV; SNtaAH, total ADHD score of teacher version of SNAP-IV; SNtaH, hyperactivity-impulsivity subscale score of teacher version of SNAP-IV; SNtaO, oppositional defiant disorder subscale score of teacher version of SNAP-IV; The *p* values are from [Mann-Whitney](https://blog.minitab.com/blog/adventures-in-statistics/best-way-to-analyze-likert-item-data%3A-two-sample-t-test-versus-mann-whitney) test result.

**Supplementary Table S9: The changes in clinical measures of the intermediate EPA tertile group after 12 weeks**

| Mean (SD) | EPA | Placebo |  |
| --- | --- | --- | --- |
| Parent Reports | (n=18) | (n=12) | *P* |
| SNpA | -3.17(6.76)- | -5.33(5.91) | .285 |
| SNpH | -2.06(4.75) | -5.08(4.54) | .072 |
| SNpO | -1.39(5.20) | -4.08(6.30) | .249 |
| SNpAH | -5.22(9.50) | -10.42(9.10) | .072 |
| Teacher Reports | (n=16) | (n=11) | *P* |
| SNtaA | -.19(1.83) | -.18(1.66) | .942 |
| SNtaH | .19(1.83) | -.27(2.72) | .422 |
| SNtaO | -1.38(4.22) | .27(2.20) | .251 |
| SNtaAH | .00(3.08) | -.45(3.14) | .645 |
| Youth Self-Reports | (n=12) | (n=10) | *P* |
| SNsA | -3.00(4.000 | -2.00 | 1.000 |
| SNsH | -3.40(4.72) | -6.00 | .667 |
| SNsO | -3.40(4.72) | -6.00 | .667 |
| SNsAH | -11.20(5.85) | -5.00 | .325 |
| SDQ | (n=18) | (n=12) | *P* |
| SDQE | -.83(1.92) | -1.25(2.56) | .819 |
| SDQC | -.89(1.53) | -1.17(1.11) | .692 |
| SDQH | -1.28(1.78) | -1.58(1.83) | .692 |
| SDQP | .00(1.46) | -.50(1.00) | .415 |
| SDQPB | .61(1.82) | .17(1.64) | .545 |
| SDQEx | -2.17(2.380) | -2.78(2.63) | .465 |
| SDQIn | -.83(2.79) | -1.75(2.30) | .545 |

Note: EPA, eicosapentaenoic acids; intermediate EPA, .91% < EPA > 1.08%; n, number; SD, standard deviation; SDQ, Strength and Difficulty Questionnaire; SDQE, SDQ Emotion; SDQC, SDQ Conduct; SDQH, SDQ Hyperactivity; SDQP, SDQ Peer Problems; SDQPB, SDQ Prosocial Behaviour; SDQEx, SDQ Externalizing Problems; SDQIn, SDQ Internalizing Problems;SNAP-IV, Swanson, Nolan and Pelham (SNAP) Questionnaire-Fourth Edition; SNpA, inattentive subscale score of parent version of SNAP-IV; SNpAH, total ADHD score of parent version of SNAP-IV; SNpH, hyperactivity-impulsivity subscale score of parent version of SNAP-IV; SNpO, oppositional defiant disorder subscale score of parent version of SNAP-IV; SNsA, inattentive subscale score of youth version of SNAP-IV; SNsAH, total ADHD score of youth version of SNAP-IV; SNsH, hyperactivity-impulsivity subscale score of youth version of SNAP-IV; SNsO, oppositional defiant disorder subscale score of youth version of SNAP-IV; SNtaA, inattentive subscale score of teacher version of SNAP-IV; SNtaAH, total ADHD score of teacher version of SNAP-IV; SNtaH, hyperactivity-impulsivity subscale score of teacher version of SNAP-IV; SNtaO, oppositional defiant disorder subscale score of teacher version of SNAP-IV; The *p* values are from [Mann-Whitney](https://blog.minitab.com/blog/adventures-in-statistics/best-way-to-analyze-likert-item-data%3A-two-sample-t-test-versus-mann-whitney) test result.

**Supplementary Table S10: The changes of the clinical measures of the high EPA tertile group after 12 weeks**

| Mean (SD) | EPA | Placebo |  |
| --- | --- | --- | --- |
| Parent Reports | (n=14) | (n=13) | *P* |
| SNpA | -.57(4.24) | -5.69(5.69) | .007** |
| SNpH | -1.43(4.18) | -5.00(6.25) | .141 |
| SNpO | -2.00(4.24) | -4.85(4.20) | .094 |
| SNpAH | -2.00(6.82) | -10.69(11.53) | .033* |
| Teacher Reports | (n=14) | (n=12) | *P* |
| SNtaA | .21(3.38)) | -3.08(3.29) | .046* |
| SNtaH | -2.42(4.18) | -2.33(2.87) | .667 |
| SNtaO | 0.00(2.91) | -1.25(4.71) | .494 |
| SNtaAH | -2.21(6.02) | -5.42(4.94) | .374 |
| Youth Self-Reports | (n=3) | (n=4) | *P* |
| SNsA | -6.00(5.58) | -3.00(5.03) | .629 |
| SNsH | -4.00(3.46) | -5.50(4.93) | 1.000 |
| SNsO | -2.67(2.30) | -5.50(3.11) | .400 |
| SNsAH | -10.00(7.55) | -8.50(8.81) | .857 |
| SDQ | (n=14) | (n=13) | *P* |
| SDQE | .07(1.33) | -1.00(1.41) | .076 |
| SDQC | -1.07(1.54) | -1.00(1.29) | .830 |
| SDQH | -.07(2.40) | -1.15(1.77) | .116 |
| SDQP | .00(2.45) | -1.23(1.54) | .259 |
| SDQPB | .21(1.72) | .15(1.34) | 1.000 |
| SDQEx | -1.14(3.28) | -2.15(2.51) | .325 |
| SDQIn | .07(2.56) | -2.23(2.20) | .038* |

Note: EPA, eicosapentaenoic acids; high EPA tertile, EPA > 1.08%; n, number; SD, standard deviation; SDQ, Strength and Difficulty Questionnaire; SDQE, SDQ Emotion; SDQC, SDQ Conduct; SDQH, SDQ Hyperactivity; SDQP, SDQ Peer Problems; SDQPB, SDQ Prosocial Behaviour; SDQEx, SDQ Externalizing Problems; SDQIn, SDQ Internalizing Problems;SNAP-IV, Swanson, Nolan and Pelham (SNAP) Questionnaire-Fourth Edition; SNpA, inattentive subscale score of parent version of SNAP-IV; SNpAH, total ADHD score of parent version of SNAP-IV; SNpH, hyperactivity-impulsivity subscale score of parent version of SNAP-IV; SNpO, oppositional defiant disorder subscale score of parent version of SNAP-IV; SNsA, inattentive subscale score of youth version of SNAP-IV; SNsAH, total ADHD score of youth version of SNAP-IV; SNsH, hyperactivity-impulsivity subscale score of youth version of SNAP-IV; SNsO, oppositional defiant disorder subscale score of youth version of SNAP-IV; SNtaA, inattentive subscale score of teacher version of SNAP-IV; SNtaAH, total ADHD score of teacher version of SNAP-IV; SNtaH, hyperactivity-impulsivity subscale score of teacher version of SNAP-IV; SNtaO, oppositional defiant disorder subscale score of teacher version of SNAP-IV; The *p* values are from [Mann-Whitney](https://blog.minitab.com/blog/adventures-in-statistics/best-way-to-analyze-likert-item-data%3A-two-sample-t-test-versus-mann-whitney) test result.

**Supplementary Table S11: The changes in biological measures of the low EPA tertile group after 12 weeks**

| Mean (SD) | EPA (n=13) | Placebo (n=16) | *P* |
| --- | --- | --- | --- |
| AA (%) | .31(3.82) | 1.37(5.04) | .268 |
| DHA (%) | .62(1.57) | .55(2.16) | .746 |
| EPA (%) | 1.06(1.32) | .13(.39) | .025* |
| Total n-3 (%) | 1.69(2.47) | .52(2.58) | .142 |
| Total n-6 (%) | .29(7.72) | 1.41(10.65) | .746 |
| N-6/n-3 ratio | -.89(2.66) | -.26(1.27) | .108 |
| hs-CRP (mg/L)  (n=13 vs 15) | -.05(0.38) | -.04(0.27) | .650 |
| BDNF (pg/mL)  (n=13 vs n=16) | -711.69 (278.24) | -908.06 (407.71) | .288 |

Note: AA, arachidonic acid; ADHD, attention deficit hyperactivity disorder; BDNF, brain-derived neurotrophic factor; DHA, docosahexaenoic acid; EPA, eicosapentaenoic acid; hs-CRP, high-sensitivity c-reactive protein; low EPA tertile, EPA < .91%; L, litre; mg, milligram; ml, millilitre; n, number; n-3, omega-3 polyunsaturated fatty acids; n-6, omega-6 polyunsaturated fatty acids; pg, picogram; SD, standard deviation. * indicates a statistical significance of *p* < .05. The *p* values are from [Mann-Whitney](https://blog.minitab.com/blog/adventures-in-statistics/best-way-to-analyze-likert-item-data%3A-two-sample-t-test-versus-mann-whitney) test result.

**Supplementary Table S12: The changes in biological measures of the intermediate EPA tertile group after 12 weeks**

| Mean (SD) | EPA (n=18) | Placebo (n=12) | *P* |
| --- | --- | --- | --- |
| AA (%) | -.70(2.42) | -1.05(3.36) | .917 |
| DHA (%) | -.34(1.27) | -.55(1.69) | .755 |
| EPA (%) | .88(.95) | -.11(.47) | .007** |
| Total n-3 (%) | .22(1.83) | -.96(2.30) | .059 |
| Total n-6 (%) | -2.80(4.95) | -2.59(6.08) | .631 |
| N-6/n-3 ratio | -.53(1.37) | 1.59(3.89) | .031* |
| hs-CRP (mg/L)  (n=18 vs 9) | -.01(.29) | -.50(1.28) | .527 |
| BDNF (pg/mL)  (n=18 vs n=10) | -656.17 (362.86) | -845.80 (351.05) | .160 |

Note: AA, arachidonic acid; ADHD, attention deficit hyperactivity disorder; BDNF, brain-derived neurotrophic factor; DHA, docosahexaenoic acid; EPA, eicosapentaenoic acid; hs-CRP, high-sensitivity c-reactive protein; intermediate EPA tertile, 91% < .EPA < 1.08%; L, litre; mg, milligram; ml, milliliter; n, number; n-3, omega-3 polyunsaturated fatty acids; n-6, omega-6 polyunsaturated fatty acids; pg, picogram; SD, standard deviation. * indicates a statistical significance of *p* < .05; ** indicates a statistical significance of *p* < .01. The *p* values are from [Mann-Whitney](https://blog.minitab.com/blog/adventures-in-statistics/best-way-to-analyze-likert-item-data%3A-two-sample-t-test-versus-mann-whitney) test result.

**Supplementary Table 13 The changes in biological measures of the high EPA tertile group after 12 weeks**

| Mean (SD) | EPA (n=14) | Placebo (n=13) | *P* |
| --- | --- | --- | --- |
| AA (%) | -1.57(2.82) | -3.22(3.32) | .325 |
| DHA (%) | -.45(1.54) | -1.60(1.32) | .048* |
| EPA (%) | .34(1.07) | -.55(.77) | .029* |
| Total n-3 (%) | -.31(2.48) | -2.45(2.08) | .081 |
| Total n-6 (%) | -2.38(4.56) | -4.95(5.88) | .220 |
| N-6/n-3 ratio | .08(1.34) | 1.88(2.44) | .113 |
| hs-CRP (mg/L)  (n=12 vs 13) | -.10(0.23) | .10(.96) | .247 |
| BDNF (pg/mL)  (n=13 vs n=12) | -686.85 (398.95) | -708.67 (362.89) | .810 |

Note: AA, arachidonic acid; ADHD, attention deficit hyperactivity disorder; BDNF, brain-derived neurotrophic factor; DHA, docosahexaenoic acid; EPA, eicosapentaenoic acid; high EPA tertile, EPA > 1.08%; hs-CRP, high-sensitivity c-reactive protein; L, litre; mg, milligram; ml, milliliter; n, number; n-3, omega-3 polyunsaturated fatty acids; n-6, omega-6 polyunsaturated fatty acids; pg, picogram; SD, standard deviation.* indicates a statistical significance of *p* < .05. The *p* values are from [Mann-Whitney](https://blog.minitab.com/blog/adventures-in-statistics/best-way-to-analyze-likert-item-data%3A-two-sample-t-test-versus-mann-whitney) test result.
